# Supplementary material for: Obesity under full fresh fruit and vegetable access conditions
Source: PLoS One. 2021 Apr 21;16(4):e0249333. doi: 10.1371/journal.pone.0249333 (PMC8059844; doi:10.1371/journal.pone.0249333)
Supplement: Attachment — Submitted filename: Plos Reviewer.pdf [file pone.0249333.s001.pdf]

Thank you very much for the positive tone of your review. We have provided an answer to each one of the aspects mentioned.

1. The authors now use an indicator variable for FFV sellers to explain BMI. However, the idea of the paper is that FFV sellers have better access to FFC products, which may affect their FFV consumption, and therefore, BMI. So, instead of estimating a reduced form model, as they do now, they should estimate a mediation model which would answer to three questions: 1) Is better access to FFV linked with FFV consumption (based on Table 2, you have information on FFV consumption), 2) Is FFV consumption associated with BMI, and 3) does FFV consumption mediate the potential link between access to FFV and BMI. Using a mediation model with adequate control variables (e.g. education, income, and sex), you could do that (see e.g. sgmediation command in STATA).

If you find that being an FFV seller is linked with higher consumption of FFV products (with adequate controls; results in Table 2 do not control e.g. for education and income), that may also support your argument that FFV sellers have better access to FFV products (R1 seemed to be concerned whether FFV sellers actually have better access).

Now the authors find, based on OLS and matching results (I am quite skeptical about your IV results, I will explain below), that being an FFV seller is associated with higher BMI, which is a bit surprising and needs an explanation. I think that with a mediation model they maybe could find an explanation to this finding.

Answer: Thank you. We have replaced the OLS results (including IV OLS) by the mediation results. The mediation results are also consistent with the matching results. FFV sellers, despite having easy access to FFV, have a higher weight and BMI. As expected, higher education and higher income are associated with lower weight and BMI.

2. Height as an outcome variable. I think this is not a relevant outcome variable in this paper. Instead of using it as an outcome variable, I would use it as a control variable in the models that use weight as the outcome variable.

Answer: Thank you. We have done it that way. We agree that it makes sense to include height as an independent variable in weight models.

3. I am skeptical of the IV results. The authors use the mother's years of educations and its square as instruments for a child's education. Intuitively, the same unobservable factors may explain both mother's and child's education years, which violates the IV assumptions. Also, Sargan's test of overidentifying restrictions (Table 8) rejects the null hypothesis (p-value in the BMI equation, i.e. in your main results, was 0.043), which implies that the instruments (as a group) are not exogenous. I would not report these results in the paper.

Answer: Thank you. We have replaced the IV OLS estimation by the mediation model results.

4. Please be explicit with your data description in section 2.2: What year(s) does your data (i.e. the data that you use in the estimations) cover? Which variables are self-reported and which are based on measurements conducted by health care professionals? For example, is BMI self-reported or not?

Answer: Thank you for this comment. We modified the “Data” subsection including the points requested. Specifically, we included: survey year used in our analyses, self-reported variables and those variables measured by health care professionals.

5. There is a detailed description of the matching model but no discussion about IV method and instrument validity. However, as said, I am skeptical that your instrument is valid so, I would drop those results.

Answer: Thank you. We have replaced the IV OLS estimation by the mediation model results.

6. In Table 2 you use t-test to indicate whether the differences are statistically significant. Why don’t you also show similar results in Table 1?

Answer: Thank you. We have included this analysis.

7. On page 7, the authors state: “the variable FFV seller shows significant effects on weight and height that may explain the weak effect on BMI”. I don’t understand this statement.

Answer:  Thanks for this comment. We have deleted the regression on height as suggested, so the phrase mentioned in this comment was also deleted.

8. The authors conclude that “years of education, more than household income, is associated with BMI reductions”. I don’t see where this interpretation comes from.

Answer:  Thank you. We have changed this phrase to incorporate the importance of both high education levels and high income levels as BMI reducers.

9. In the Discussion section: “We used the ENS in Chile to assess the change on FFV sellers’ obesity related indicators”. The way I understood the setup, the authors explain BMI levels, not changes.

Answer: That was a mistake, thanks for pointing at it.

10. Table 3: In column 3, the authors use population weights. If sample weights are necessary, why don’t you use them in the OLS and matching models? Also, if weights are necessary, please explain that in the section “Materials and Methods”.

Answer: Thank you. We have dropped the population weights from the estimation. We used them only for the descriptive statistics.

11. On page 3 (last paragraph), there are two times “second” (should be second and third). Therefore it is unclear to which point the authors refer when they say that “we cannot test the second condition directly”.

Answer: Yes, that was a mistake. Thank you for catching it. Now it says third instead of second. We have also eliminated that a condition could not be tested, as all of them could eventually be tested for. However, it seems there isn’t a validated test for unconfoundedness available yet.  We do show supporting evidence for conditions one (overlap) and two (balanced sample) in the Appendix.

12. We have rewritten equation (2) as we noticed that we had the ATT instead of the ATE.
Old equation:
\tau=E[E(Y_{1i}|D_i=1,p(X_i))-E(Y_{0i}|D_i=0,p(X_i))|D_i=1]

New equation:
\tau=E(Y_{1i}|D_i=1,p(X_i))-E(Y_{0i}|D_i=0,p(X_i))
