# Supplementary material for: Obesity under full fresh fruit and vegetable access conditions
Source: PLoS One. 2021 Apr 21;16(4):e0249333. doi: 10.1371/journal.pone.0249333 (PMC8059844; doi:10.1371/journal.pone.0249333)
Supplement: Attachment — Submitted filename: Reviewers.pdf [file pone.0249333.s002.pdf]

PONE-D-20-02163R2
Obesity under Full Fresh Fruit and Vegetable Access Conditions
PLOS ONE

Answers to Reviewer #2

1) Third sentence of the abstract should read, "Using fruit and vegetable access as a mediator, we found that years of education and household income are correlated with a decrease in obesity." (Add "access" and remove comma.)
2) Line 13; "agricultural farmers" is redundant. Just say "farmers." I think this occurred in one other place as well.
3) Sentence on lines 70 - 72 is unclear. ". . . socio-demographic characteristics lead to a food consumption pattern or the opposite." Unclear what "opposite" means in this context.
4) Lines 115 and 275 - comma is unnecessary
5) Table 2 - acceptable abbreviation for grams is "g" not "gr"
6) Table 3 - "height" is misspelled
7) Line 294 - "to" should be "with"

Authors: Thank you. We have changed all the above comments made by the reviewer.

The authors are to be congratulated on a fine paper.

Authors: Thank you. We appreciate the constructive feedback over the review process.

PONE-D-20-02163R2
Obesity under Full Fresh Fruit and Vegetable Access Conditions
PLOS ONE

Answers to Reviewer #3

1. In the introduction, the authors refer to the results from the mediator model solely saying "using mediation regression, we found that years of education and household income are associated with a smaller BMI". This is a bit odd because this is not the main point why you performed a mediation analysis.

Authors: Thanks for pointing at that. We have edited the paragraph to make it more informative (the changes are highlighted in the text).

2. Mediation model: The authors refer to Baron & Kenny, who suggest that mediation analysis should be done in three steps. The paper by Iacobucci et al. (2007, p.153), to which the authors also refer, proposes conducting the mediation analysis via SEM. Whether mediation is significant is typically also tested using e.g., the Sobel test. It seems that this is not what the authors have done. In Table 3, they estimate two regressions separately.

Authors: In the lines 255 to 259, we have added an additional explanation regarding the estimation procedure and the Sobel test results are available in the appendix.

3. Interpretation of the mediation results: The idea of the mediation model is to see 1) whether the independent variable (being an FFV seller) is associated with the mediator (FV consumption); 2) whether the mediator (FV consumption) is associated with the outcome (BMI) and; 3) whether this mediated pathway (1 and 2) is significant. The "FFV seller" coefficients in Table 3 (Columns 2 and 4) are related to point 1, and the "FV portions" coefficients in Table 3 (Columns 3 and 5) are related to point 2. The authors do not comment at all the aforementioned "FFV seller" coefficients in columns 2 and 4. Then, based solely on the "FV portions" coefficient, the authors conclude that "FV consumption is not acting as a mediator to explain BMI variation". In some sense that applies, because for the mediation pathway to be significant, there needs to be a significant relationship between the independent variable and mediator and between mediator and outcome variable. However, making this interpretation solely based on the association between mediator and outcome variable is odd. E.g., the Sobel test could be used to test the significance.

Authors: We recognize that the previous discussion needed to be refined. In the line 255 to 259, we have added further discussion and support based on the Sobel test. As presented in the Appendix, the Sobel test results are aligned with our results of no mediation path through FV consumption.

4. Findings: The authors find that FFC sellers have higher BMI/weight. Why? It is hard for me to understand why simply being an FFV seller is associated with higher BMI unless there are unobserved confounders that affect the results. One potential explanation for this finding would have been that because FFV sellers have better access to FV products, they consume more calories (i.e., they consume FV products on top of all other food products). However, the mediation model results imply that this is not the case: FFV sellers do not seem to consume more FV products, and FV product consumption does not seem to be associated with BMI.

Authors: Thank you for pointing at that. Based on our analysis, fresh FV sellers have a weight and BMI similar to people with the same education level. Columns 4 and 5 in Table 3 show that weight and BMI are not significantly different comparing fresh FV sellers and the rest of the population when using a low-education subsample. Therefore, the variations on weight and BMI are associated with changes on education rather than with being a fresh FV seller. We understand that we need to make this more explicit in the discussion section.

5. Conclusions: The main finding from the mediation model should be stated more clearly. Based on the results, the main finding seems to be that having better FFV access does not reduce BMI because better access is not associated with FFV consumption and because FFV consumption is not associated with BMI. There is no discussion about the finding that FFV consumption is not associated with weight. Does this finding receive support from other studies? From the point of view that FFV consumption is not associated with weight, some parts in the "Discussion" section seem a bit odd.

Authors: Thank you. We have incorporated your comments in the discussion section and stated the mediation results more explicitly. We also included a discussion on our finding relating FV consumption and weight and added references that support our findings.

6. Causal terminology: The authors use causal terminology (e.g., "the fifth column shows the effect on weight…"), although the methods they use are usually not considered to allow causal interpretation.

Authors: Thank you for this comment. We did our best to eliminate any causal interpretations/terminology of our results.

7. Limitations of the study: The limitations of the study and the implications of these limitations require more discussion. For example, 1) the number of FFV sellers is rather small (96). This is a bit unfortunate because there is some indication that FFV sellers consume more FV products, but the differences are not significant. 2) Although the authors argue otherwise, it is possible that FFV sellers actually do not have better access to FV products. The argument that FFV sellers have better access to FV products, cannot

be tested directly. If the authors would have found that FFV sellers consumed more FV products, that might have supported their argument. 3) Potential confounders (see my previous point 4).

Authors: Yes, thank you for this comment. We have expanded the limitations' paragraph considering your suggestions. We did not mention potential confounders explicitly in the limitations as we have already said that having cross sectional data is a limitation and we believe this implies potential omitted variables that could be correlated with independent and dependent variables. Along these lines, we also added that the survey was not originally designed to measure fresh FV sellers' lifestyle.

8. At some point, I got lost with abbreviations FFV and FV. The authors talk e.g., about FFV access and FFV baskets but also about FV consumption. Would one abbreviation be enough?

Authors: Point taken. We have kept "FV". When we need to refer to fresh fruit and vegetables, we indicate "fresh FV".

Thank you for your comments.
