# Supplementary material for: Obesity under full fresh fruit and vegetable access conditions
Source: PLoS One. 2021 Apr 21;16(4):e0249333. doi: 10.1371/journal.pone.0249333 (PMC8059844; doi:10.1371/journal.pone.0249333)
Supplement: Attachment — Submitted filename: Reviewers.pdf [file pone.0249333.s003.pdf]

PONE-D-20-02163R3
Obesity under Full Fresh Fruit and Vegetable Access Conditions
PLOS ONE

Answers to Reviewer #3

I still have major concerns regarding the paper, particularly regarding the mediation analysis.

1. The main results should be presented more clearly throughout the paper (abstract, intro, results, and conclusions).

Authors: Thanks for pointing at that. We have edited the four sections that you mentioned to highlight the results and explain them more clearly (the changes are highlighted in the text).

2. The authors do not explain in the paper how the mediated path is obtained/calculated.

Authors: Thank you. We have incorporated an explanation in the Methodology section and we explain the computation in the Results section (the changes are highlighted in the text).

3. I do not understand Table 5. Based on Table 3, the indirect effect in the weight model is (0.397*0.0291) 0.021. However, based on Table 5 it is 0.000. You can do the Sobel test using the delta method. Why are there separate columns for Sobel and delta? Based on table notes the standard errors in all columns (delta, sobel, monte carlo) are based on bootstrapping?

Authors: We have corrected the Stata code. Now, Tables 3 and 5 present unstandardized coefficients, therefore, both tables are consistent (for weight equation: 0.3971*0.0291=0.012). To the best of our knowledge, Delta Method can be used or not to obtain the standard errors, which explain the two columns. Now, to avoid confusion, we have chosen to drop the Delta Method column, whose results are very similar to the other two test results. We have corrected the table notes, the bootstrapping is only for the Monte Carlo approach.

4. Tables should be self-standing. In Table 4, it is unclear to which variable the coefficients refer to.

Authors: Thanks for pointing at that. We have edited the footnote of Table 4 to make it more self-standing.

5. The first paragraph of section 2 (page 3): I do not think the description of the mediation model really captures the essence of the method.

Authors: Yes, thank you for this comment. We have edited the explanation including two references. The explanation of the mediation model is at the beginning of section 2 and continues in section 2.2.

6. The second paragraph in section 2.2: "Fresh FV sellers' access can directly affect BMI…" I would put this just "being a fresh FV seller". Access to me seems to refer to the mediation pathway.

Authors: Thanks for pointing at that. We have corrected it (the changes are highlighted in the text).

7. The authors should be more careful with causal terminology. E.g., "We found that having better fresh FV access does not reduce BMI". "… education leads to a stronger effect…"

Authors: Point taken. We have reviewed the causal methodology in the full article and hope to have eliminated all the causal terminology (the changes are highlighted in the text).

8. Relative to the methods and findings of this paper, I find the conclusion that "education needs to be part with a more comprehensive public policy" a bit excessive.

Authors: We recognize that the previous statement was too strong. We have softened it. (The changes are highlighted in the text).

9. I did not understand the following sentence in the Discussion section: "Since our results show that fresh FV sellers consume similar amounts of FV compared to the rest of the population, we do not have direct evidence… …. who consume more FV and have lower BMI)."

Authors: Thanks for pointing at that. We have eliminated the statement (the changes are highlighted in the text).

10. As a limitation, the authors mention that FV sellers could understate their true income. How is that a major limitation?

Authors: We believed that as income is one of our control variables, and it is not directly observed, we cannot completely rely on how accurate it is. However, we understand that, following the previous comment, sample size rather than income measurement is a major limitation in this case. Therefore, we have decided to drop this statement to make this section more straightforward/accurate.
